# Reduced anterior insular cortex volume in male heroin addicts: a postmortem study

**DOI:** 10.1007/s00406-023-01553-6

**Published:** 2023-01-31

**Authors:** Ulf J. Müller, Lucas J. Schmalenbach, Henrik Dobrowolny, Paul C. Guest, Konstantin Schlaaff, Christian Mawrin, Kurt Truebner, Bernhard Bogerts, Tomasz Gos, Hans-Gert Bernstein, Johann Steiner

**Affiliations:** 1 Department of Psychiatry and Psychotherapy, University of Magdeburg, Magdeburg, Germany; 2 Translational Psychiatry Laboratory, University of Magdeburg, Magdeburg, Germany; 3 Forensic Psychiatric State Hospital of Saxony-Anhalt, Stendal-Uchtspringe, Germany; 4 Laboratory of Neuroproteomics, Department of Biochemistry and Tissue Biology, University of Campinas (UNICAMP), Campinas, Brazil; 5 Department of Neuropathology, University of Magdeburg, Magdeburg, Germany; 6 Center for Behavioral Brain Sciences, Magdeburg, Germany; 7 Institute of Legal Medicine, University of Duisburg-Essen, Essen, Germany; 8 Salus Institute, Magdeburg, Germany; 9 Department of Forensic Medicine, Medical University of Gdańsk, Gdańsk, Poland; 10 German Center for Mental Health (DZP), Center for Intervention and Research On Adaptive and Maladaptive Brain Circuits Underlying, Mental Health (C-I-R-C), Jena-Magdeburg-Halle, Germany; 11 Center for Health Und Medical Prevention (CHaMP), Magdeburg, Germany

**Keywords:** Heroin, Addiction, Insula, Postmortem, Volumetry, Morphometry

## Abstract

We and others have observed reduced volumes of brain regions, including the nucleus accumbens, globus pallidus, hypothalamus, and habenula in opioid addiction. Notably, the insular cortex has been under increasing study in addiction, and a smaller anterior insula has been found in alcohol-addicted cases. Here, we have investigated whether similar effects occur in heroin addicts compared to healthy controls. Volumes of the anterior and posterior insula in heroin addicts (*n* = 14) and controls (*n* = 13) were assessed by morphometry of Nissl-myelin-stained serial whole-brain coronal sections. The mean relative volume of the anterior insular cortex was smaller than in non-addicted controls (3010 ± 614 *10^–6^ versus 3970 ± 1306 *10^–6^; *p* = 0.021**)**. However, no significant differences in neuronal cell counts were observed. Therefore, the observed volume reduction appears to be a consequence of damaged connecting structures such as neuropil and glial cells. The findings were not confounded by age or duration of autolysis. Our results provide further evidence of structural deficits in key hubs of the addiction circuitry in heroin-dependent individuals and warrant further research in this area.

**Supplementary Information:**

The online version contains supplementary material available at 10.1007/s00406-023-01553-6.

## Introduction

Heroin is considered as one of the most harmful illicit drugs in the world with a negative impact on society and public health [1]. On an individual basis, opioid addiction is a severe and potentially lethal disease. In 2020, more than 13,000 people in the US died from a drug overdose involving heroin [2] and this problem has also become significant in other world regions such as multiple European countries [3]. Furthermore, heroin has been associated with increased use during the COVID-19 pandemic [4].

In previous studies, we observed reduced volumes of brain regions including the nucleus accumbens, globus pallidus, hypothalamus, and habenula, with reduced neuronal cell counts in postmortem brains of male heroin addicts [5–8]. This provided evidence of potential structural deficits combined with the known function deficiencies caused by opioid addiction.

The human insular cortex (also known as the island of Reil or the insula) is a neocortical region hidden in the depth of the Sylvian fissure [9]. This large and heterogenous structure is surrounded by the circular sulcus of Reil and can be divided anatomically into anterior and posterior parts along the approximate border of the central insular sulcus [10]. The *anterior insula* is functionally associated with cognition, motivation and emotion, while the *posterior insula* is associated with sensory interoception [11]. The first investigation which implicated the insula as a critical neural substrate for addiction was a lesion-based study showing that insular damage disrupted addiction to cigarette smoking [12]. Another lesion study showed a similar disruption of opium addiction after cerebrovascular accidents of the insula [13]. Several neuroimaging and animal studies have now demonstrated a key role of the insular cortex in addictive behavior, especially regarding the loss of drug intake control [14], craving [15], and propensity to relapse [16].

Recently, the insula has been proposed as a potential brain stimulation target for the treatment of addiction [17]. Thus far, there has been only one human study on this, in which combined deep transcranial magnetic stimulation of the prefrontal and insular cortices resulted in reduced cigarette consumption [18]. In a rodent morphine addiction model, deep brain stimulation (DBS) of the anterior insula prevented morphine-conditioned relapse and reversed the expression of morphine-regulated proteins [19]. These results suggest that DBS of the anterior insula may represent a useful approach in the treatment of substance use disorders. Additionally, studies have identified several indices of structural changes within the insular cortex in addiction. Studies have shown that cocaine-dependent patients have decreased gray matter concentration and reduced cortical thickness of the insular cortex [20, 21], while reductions in anterior insular cortical thickness and volume have been reported in alcohol addiction [10, 22, 23]. In heroin addiction, decreases in gray matter density of the insular cortex have been found using voxel-based morphometry [24, 25].

In the current study, we have tested the hypothesis that structural deficits also occur in the insula of heroin addicts. Similar to our previous studies, we have assessed this using measurements of both insular volumes and neuronal cell counts in paraffin-embedded whole brain sections.

## Materials and methods

### Subjects

All brains were obtained from the Magdeburg Brain Bank. Sampling and preservation of the human brain material were done in accordance with the Declaration of Helsinki, German law and the local institutional review board at the University of Magdeburg. The analysis included 14 chronic male heroin addicts who died from drug overdose and 13 male controls (Table 1). Patients and controls were matched as closely as possible but differed in terms of age and duration of autolysis (although the latter did not reach significance; *p* = 0.097). Information on clinical characteristics was extracted from the clinical records and via structured interviews with people closely related to the subjects using a psychological autopsy [26]. In addition to heroin, all but one of the addicts had a history of abusing other legal and/or illegal substances, including morphine, cannabis, alcohol, cocaine, barbiturates, benzodiazepines and hallucinogens. However, the tested patients fulfilled the diagnostic criteria of an addiction only for heroin. An experienced neuropathologist (CM) ruled out qualitative neuropathological changes due to neurodegenerative disorders (such as Alzheimer’s disease, Parkinson’s disease, Pick’s disease), tumors, or inflammatory, vascular or traumatic processes, using samples with Nissl myelin staining, as well as HLA-DR-, beta-amyloid-, and tau-immunostainings. None of the heroin addicts were HIV-positive. A toxicology screen of blood and urine for ethanol and other substances of abuse was performed at each medico-legal autopsy and evaluated by forensic pathologists (KT and TG).

**Table 1:** Demographic, relative anterior and posterior insular volumes and neuronal cell count data of patients with heroin addiction (*n* = 14) and healthy control subjects (*n* = 13)

	Heroin addict (*n* = 14)	Control (*n* = 13)	Statistical test	Test value	*p* value	Effect size (Cohen’s *d*)
Demographic parameter						
Age (years)	30.86 ± 7.57	44.85 ± 10.41	*t* test	*T* = − 3.968	< 0.001***	− 1.547
Duration of autolysis (hours)	52.57 ± 44.19	30.38 ± 16.12	*t* test	*T* = 1.757	0.097	0.657
Volume shrinkage factor (VSF)	1.758 ± 0.310	1.970 ± 0.448	*t* test	*T* = − 1.416	0.171	− 0.553
Total brain volume (cm^3^)	1534 ± 65	1390 ± 104	*t* test	*T* = 4.287	< 0.001***	1.679
Relative anterior insula volume * 10^–6^ (mm^3^/mm^3^)						
Total *10^–6^ (mm^3^/mm^3^)	3010 ± 614	3970 ± 1306	rmANOVA	*F* = 6.112	0.021*	− 0.989
> Left *10^–6^ (mm^3^/mm^3^)	1495 ± 305	1998 ± 659	Post-hoc	*F* = 6.638	0.016* (FDR: *p* = 0.030*)	− 1.031
> Right *10^–6^ (mm^3^/mm^3^)	1515 ± 329	1972 ± 660	Post-hoc	*F* = 5.295	0.030* (FDR: *p* = 0.030*)	− 0.921
Relative posterior insula volume *10^–6^ (mm^3^/mm^3^)						
Total *10^–6^ (mm^3^/mm^3^)	1465 ± 384	1604 ± 585	rmANOVA	*F* = 0.542	0.469	− 0.294
> Left *10^–6^ (mm^3^/mm^3^)	749.7 ± 249.8	778.9 ± 262.0	Post-hoc	*F* = 0.088	0.769 (FDR: *p* = 0.769)	− 0.119
> Right *10^–6^ (mm^3^/mm^3^)	714.8 ± 198.5	824.6 ± 353.2	Post-hoc	*F* = 1.011	0.324 (FDR: *p* = 0.648)	− 0.402
Estimated neuron number in anterior insula * 10^–6^						
Total sum	179.07 ± 36.90	200.3 ± 81.4	rmANOVA	*F* = 0.779	0.386	− 0.353
> Left	88.70 ± 18.10	101.5 ± 40.1	Post-hoc	*F* = 1.168	0.307 (FDR: *p* = 0.511)	− 0.432
> Right	90.37 ± 20.53	98.79 ± 42.17	Post-hoc	*F* = 0.445	0.523 (FDR: *p* = 0.511)	− 0.267

All subjects were male. ANOVA and FDR-adjusted post-hoc *t* tests are presented. Statistically significant values are in bold text

Annotation: data in columns “Heroin addicts” and “Controls” are presented as mean ± standard deviation

*p < 0.05

**p < 0.01

***p < 0.001; Cohen’s *d* ≥ 0.2,  ≥ 0.5 and ≥ 0.8 were considered as small, medium and large ES

### Tissue processing

Tissue preparation was performed as previously described [27]. Brains were removed and fixed *in toto* in 8% phosphate-buffered formaldehyde for at least two months. Frontal and occipital poles were separated by coronal cuts anterior to the genu and posterior to the splenium of the corpus callosum. After embedding all parts of the brains in paraffin wax, serial, whole brain coronal sections of the middle block were cut using a large-scale microtome (Balzers, Liechtenstein) at a thickness of 20 μm and mounted. Volume shrinkage was determined for each brain before and after dehydration and embedding of the tissue using the formula: VSF = (A1/A2)^3/2^ (VSF = volume shrinkage factor; A1 = cross-sectional area before processing of tissue; A2 = cross-sectional area after processing of tissue).

### Volumetric analysis

**Fig. 1:**
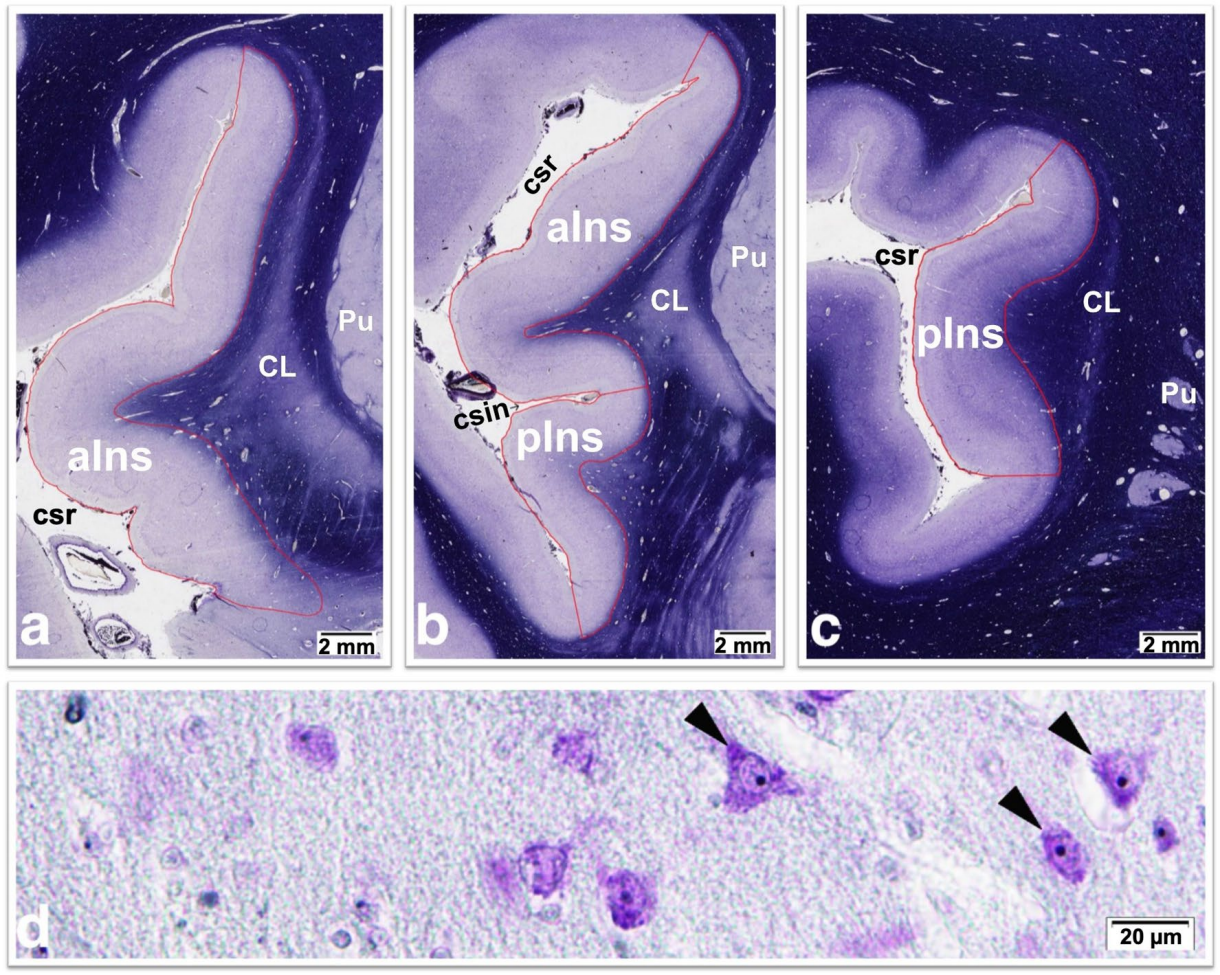
Delineation of the anterior (aIns) and posterior (pIns) insular cortex of the right hemisphere with adjacent structures in representative anterior (**a**), middle (**b**) and posterior (**c**) Nissl- and Heidenhain-Woelcke-stained sections (12.5 × magnification). *CL* claustrum, *csin* central sulcus of insula, *csr* circular sulcus of reil, *Pu* putamen. **d** Neuronal cell numbers (pyramidal cells and interneurons) in the insular cortex were determined in 200 × magnification. Representative image (cutout of counting field) with arrowheads pointing to neurons with clearly visible nucleoli

For anatomical orientation and morphometric investigations, every 50th serial coronal whole brain section was treated with a combined cell and fiber solution of Nissl (cresyl violet) and Heidenhain-Woelcke myelin stains and sampled [28, 29], resulting in an intersectional distance of 1 mm. All subsequent examinations were performed using an Olympus BX60 microscope with an associated DP22 camera and the CellSens image analysis program (OLYMPUS K.K., Tokyo, Japan), with the operators blinded to diagnosis. Overview images of the whole area of the insular complex were captured at 12.5 × magnification with a linear resolution 2.990 μm/px and an automatic exposure time. All available serial coronal brain sections (average of 31 sections per brain) with uni- or bilaterally recognizable insular cortex were evaluated. On the scanned overview images, delineation of the insular cortex was determined as described by Ding et al. [30] and as illustrated in Fig. 1. Subdivision of the insular cortex into the anterior and posterior regions was performed as described by Senatorov et al. based on the location of the central insular sulcus [10]. Using the CellSens function “freehand polygon”, the cross-sectional areas of the insula were outlined and measured by planimetry in both hemispheres. Insular cortex volumes were calculated by summing the measured areas multiplied by the distance to the next measured section, as previously described [31, 32]. These were multiplied by the VSF (related to the dehydration and embedding process) to estimate the insular volumes in fresh brain tissue.

### Neuronal cell counting

Neuron counting was performed only in the anterior insula as the main region of interest. The images were captured at 200× magnification with a linear resolution 0.18343 μm/px and a constant exposure of 9.092 ms (Fig. 1). In the acquired images, both pyramidal cells and interneurons with clearly visible nucleoli were counted manually within the delineated cross-sectional area of the anterior insular cortex. We applied 30 counting boxes for each hemisphere per section, with three sections per case, resulting in 180 counting boxes per case. Each box corresponded to a size of 0.0016742884 mm^3^ = 352.2 μm × 264.1 μm × 18 μm edge length (20 µm section thickness minus guard zones of 2 × 1 µm). The observed coefficients of error (OCE) were calculated as described by Gundersen and Jensen [29]. The mean OCE values were 0.078. The Pearson interrater correlation showed good reliability with a value of *r* = 0.872. A counting grid was used to define a three-dimensional box within the thickness of the section as described previously [27], allowing 2 × 1 μm guard zones at the top and bottom of the section for the application of a direct, three-dimensional counting method.

Cortical layer-specific evaluation was performed by subdividing cortical gray matter regions into superficial and deep layers, as proposed by Katsel et al. [33]. As summarized in Table 2, five counting boxes were placed in layers III and IV each. Layers I and II as well as V and VI were combined due to difficulties in exact delineation (10 counting boxes per double layer). Care was taken that the counting boxes did not overlap. Application of the optical disector made it necessary to measure movements in the z-axis using a microcator as an integral part of the microscope. The determined numerical densities of neurons per counting box were averaged per layer and summed. Estimated total neuron numbers of the left and right anterior insular cortex were extrapolated by multiplying the averaged numerical densities of neurons from all cortical layers with the determined respective total anterior insular cortex volumes in mm^3^ divided by 0.0016742884 mm^3^ (i.e., size of one counting box).

**Table 2:** Distribution of counting boxes across the cortical layers

Layers	Number of counting boxes
Combined layers I and II	10
Layer III	5
Layer IV	5
Combined layers V and VI	10

### Statistical analysis

Data analyses were performed using the statistical software package R (http://www.r-project.org). Demographic data were compared using Student’s *t* tests. Volume data were normally distributed as indicated by Shapiro–Wilk-tests. As summarized in Table 1, total brain volume was larger in the heroin group compared to controls (1534 ± 65 versus 1390 ± 104 cm^3^; *T* = 4.287; *p* < 0.001). In determinations of diagnosis-dependent insular cortex volumes, we accounted for whole brain size as a potential confounding factor using relative insular volumes (corrected by VSF) normalized to whole brain volume (relative volume = volume of the respective insular structure divided by the whole brain volume). For *analysis of relative insular volume data*, a repeated measures analysis of variance (rmANOVA) was performed using “hemisphere” and “region” (anterior/posterior insula) as within-subject factors and “diagnosis” as a between-subject factor. As there was a “region*diagnosis” interaction, an rmANOVA was performed for each region. To account for multiple comparisons, false discovery rate (FDR)-corrections were performed for *p* values of post-hoc *t* tests for each rmANOVA [34]. Since the *number of neurons* was determined only in the anterior insula, an overall rmANOVA for the within-subject factor “region” could not be used, but all other steps were identical as in the relative volume analysis. Apart from the main effects of “diagnosis” or “hemisphere”, the interaction of these factors was determined by rmANOVA. Cohen’s *d* was used to assess effect size (ES), with *d* ≥ 0.2,  ≥ 0.5 and ≥ 0.8 considered as small, medium and large ES, respectively.

*To identify potential confounding factors*, rmANCOVA were performed with the testwise inclusion of “age” and “duration of autolysis” as covariates. In addition, Pearson correlation coefficients were used to calculate correlations of anterior insula volumes with respective neuronal cell densities, age with anterior/posterior insula volumes, and “duration of autolysis” with the shrinkage factor. Finally, Pearson correlation coefficients were calculated to assess the potential association of anterior insula volume reduction with previously published brain volumetric data from patients with heroin addiction [5–8].

All statistical tests were two-tailed, and significance was defined as *p* < 0.05.

## Results

### Volumetric analysis

**Fig. 2:**
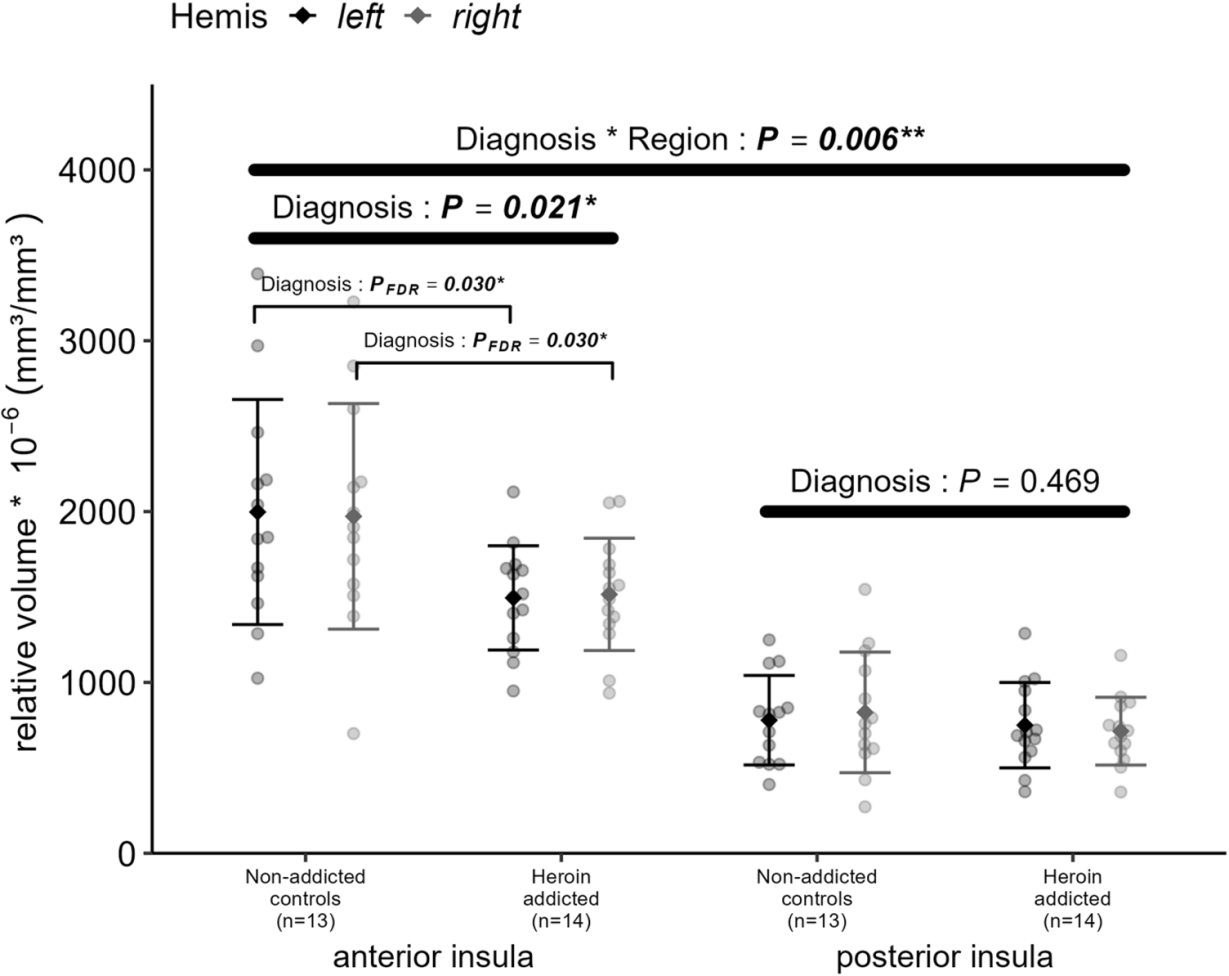
Relative volumes of the anterior and posterior insula in patients with heroin addiction compared to healthy controls. Insular volumes were normalized by total brain volume. Data are presented as mean ± standard deviation

The relative insular volume showed an interaction between diagnosis and region (anterior/posterior insula) [*F* (1,25) = 8.993, *p* = 0.006**]. However, there was no cerebral lateralization (i.e., no effect of “hemisphere”) and the factors “diagnosis” and “hemisphere” did not interact significantly. Because of the significant diagnosis*region interaction, a separate analysis of the relative volume of the anterior and posterior insula was carried out. The anterior insula showed a smaller relative volume in heroin addicts versus controls (3010 ± 614 *10^–6^ versus 3970 ± 1306 *10^–6^; *F*(1,25) = 6.112, *p* = 0.021*, Cohen’s *d* = − 0.989; Table 1 and Fig. 2). Consistent with this finding, the proportion of the anterior to total insula was also found to be lower in heroin addicts versus controls (67.50 ± 5.47% versus 71.44 ± 2.89%; *F*(1,25) = 5.345, *p* = 0.029*, Cohen’s *d* = − 0.925). No significant diagnosis-related differences were observed regarding the relative volume of the posterior insula (Table 1 and Fig. 2).

### Neuronal cell counting

Due to the above-mentioned volume reduction of the anterior insula, neuronal counting was performed to check whether this volume reduction might be associated with neuronal loss. However, relative volumes of the anterior insula showed no significant correlation with respective neuronal cell densities. Moreover, there was no main effect of “diagnosis” on estimated neuron numbers [179.07 ± 36.90 versus 200.3 ± 81.4; *F*(1,25) = 0.779, *p* = 0.386; Table 1].

### Exploration of potential confounding variables

Testwise rmANCOVAs employing potential confounding variables did not reveal a significant effect of age [anterior insula: *F*(1,24) = 0.654, *p* = 0.427; posterior insula: *F*(1,24) = 0.001, *p* = 0.972] or duration of autolysis [anterior insula: *F*(1,24) = 0.615, *p* = 0.441; posterior insula: *F*(1,24) = 0.040, *p* = 0.843] on diagnosis-dependent relative insular volume differences. In addition, the percentage of anterior to whole insula volume was not affected by age [*F*(1,24) = 0.419, *p* = 0.524) or autolysis [*F*(1,24) = 2.022, *p* = 0.168]. With neuronal cell count data available only for the anterior insula, testwise rmANCOVAs employing potential confounding variables did not reveal a significant influence of age [*F*(1,24) = 1.361, *p* = 0.255] or duration of autolysis [*F*(1,24) = 0.049, *p* = 0.827] on diagnosis-dependent neuronal cell count differences. In addition, no significant correlations were found between age and anterior insula volume (*r* = 0.280, *p* = 0.160) or between the duration of autolysis and shrinkage factor (*r* = − 0.140, *p* = 0.490).

### Correlation with previous findings

Comparison of the current findings with those from our previous studies [5–8] showed that volumes of the right anterior insula and right nucleus accumbens were correlated in heroin-addicted subjects, although this did not pass FDR correction. No further significant correlations were observed with previous volumetric findings of our workgroup on heroin addiction (Supplementary Table 1).

## Discussion

To our knowledge, this is the first study investigating volume and neuronal cell counts of the insular cortex in postmortem brains of heroin-addicted individuals. We found significantly reduced relative anterior but not posterior insula volumes and no differences in neuronal cell numbers in heroin-addicted subjects. These findings provide further evidence of the pathophysiological role of the anterior insular cortex in opioid addiction by highlighting the point that structural alterations may be associated with the known functional abnormalities in a brain region-specific manner. Therefore, this study extends previous findings by our group on reduced volumes of the nucleus accumbens, hypothalamus and globus pallidus, and both reduced volume and neuronal cell numbers in the habenula in heroin addiction [5–8].

The current finding that volumes of the right anterior insular cortex were correlated with those of the right nucleus accumbens in heroin-addicted individuals is consistent with previous studies showing structural connectivity between these brain regions in healthy subjects, as demonstrated by diffusion tensor imaging [35]. In addition, studies in rodents have shown that glutamatergic projections from the insular cortex to the core of the nucleus accumbens are required for the reinstatement of cue associated morphine seeking behavior [36], and glutamatergic inputs from the anterior insula to the nucleus accumbens are necessary for compulsive alcohol seeking [37]. Therefore, we hypothesize that structural deficits may exist in functionally connected cortical and subcortical structures in opioid addiction [35]. However, further research is needed into this potential association and its implications for the neurobiology of opioid addiction.

Several brain regions are connected to the anterior insular cortex. Network approaches postulate the anterior insula and anterior cingulate cortex (ACC) together as key nodes of a large-scale brain network, the salience network (SN), that coordinates resources between the default mode network and the central executive network and initiates network switching [38, 39]. Abnormal functional connectivity and network switching are thought to play a role in addiction [40]. Resting-state functional connectivity (rsFC) studies showed weaker rsFC of the insular cortex and ACC in opioid addicts, implicating a dysfunctional SN [41]. Abstinent heroin users showed increased rsFC of the insula and amygdala, supporting the hypothesis that the insula plays a role in drug-seeking through increased synchronization with the amygdala, contributing to the loss of control and impaired inhibitory behavior [42].

Several magnetic resonance imaging (MRI) studies have found volume reductions of the insular cortex in opioid addiction and other substance use disorders [43–45]. Similar to our findings, a decreased volume of the anterior insular cortex but not of the posterior insular cortex has been reported in alcoholism [10, 23]. Complementing their MRI study, Senatorov et al. analyzed postmortem brains of 6 alcohol-dependent and 6 control subjects and found that the total number of neurons did not differ, in agreement with our study [10].

Because there is evidence that heroin has neurotoxic effects [46, 47], it is conceivable that the observed volume deficit in the anterior insula could be the result of chronic heroin use. This is partially contradicted by the fact that we did not detect significant diagnosis-related changes in the number of neuronal cells in this cortical area. Moreover, our samples had been investigated microscopically by an experienced neuropathologist (CM), who did not observe neurodegenerative changes. Therefore, the observed volume reduction may be a consequence of damaged connecting structures such as neuropil and glial cells. For example, in schizophrenia, the loss of volume in the dorsolateral prefrontal cortex is probably due to a reduction in the number of astrocytes and oligodendrocytes, as is the reduction in hippocampal volume in depression and schizophrenia [48–51]. However, there is a lack of volumetric studies focusing on glial cells, especially in addiction research. Although toll-like receptor-mediated microglial cell activation [52] and a consecutive proinflammatory phenotype caused by opioid administration has been found previously [53, 54], microglial activation has not been shown in the insular cortex until now. This highlights the need for more histopathological research in this brain region in studies of addiction.

It is also possible that the structural abnormalities of the anterior insula may be related to a higher vulnerability to addiction. Insular volumes have been shown to be lower in other neuropsychiatric diseases such as mood disorders [55] and schizophrenia [56, 57], and addictions and mood disorders are often comorbid [58]. While in the current study, there is no evidence of mood disorders in the clinical records, we cannot rule out the possibility that the typical clinical features of these conditions might have been obscured by the psychopathology associated with addiction. Although our results of decreased anterior insula volume in heroin addiction suggest that this may be a substrate of disturbed function, it remains to be elucidated whether this effect precedes addiction and represents a vulnerability, or if it is a consequence of the neurotoxic effects of repeated heroin exposure or both.

It should be noted that this study is potentially limited by several factors. As with any postmortem analysis, no longitudinal data was obtained. Due to the limited clinical records, there are no reliable data on the duration of individual addiction or on cumulative heroin use. Therefore, we cannot analyze whether these two factors played a significant role or if a reduced volume might predispose to addiction itself. In addition, the sample size of this study was relatively small and our brain bank only contains the brains of male addicts. Also, we cannot rule out the potential effect of other drug use on the findings. However, this is not likely as addiction criteria were fulfilled only for heroin. Finally, heroin addicts were significantly younger than controls and showed a non-significant trend towards a higher duration of autolysis. However, testwise rmANCOVAs revealed no significant influence of either of these factors on volume and neuronal cell count data.

In conclusion, the present results provide further evidence of structural deficits in key hubs of the addiction circuitry in heroin-dependent individuals. We found that the relative volumes of the anterior insular cortex were smaller in male addicts compared to controls but no significant differences in neuronal cell counts were observed. Therefore, the observed volume reduction appears to be a consequence of damaged connecting structures such as neuropil and glial cells. We propose that future studies of this brain regional volume change in heroin addiction should focus on these and other components associated with neuronal connectivity.

## Supplementary Information

Below is the link to the electronic supplementary material.

Supplementary file1 (DOCX 40 KB)

## Data Availability

The data that support the findings of this study are available from the corresponding author upon reasonable request.
