# Supplementary material for: Reduced anterior insular cortex volume in male heroin addicts: a postmortem study
Source: Eur Arch Psychiatry Clin Neurosci. 2023 Jan 31;273(6):1233–41. doi: 10.1007/s00406-023-01553-6 (PMC9888352; doi:10.1007/s00406-023-01553-6)
Supplement: Supplementary file 1 — Supplementary file1 (DOCX 40 KB) [file 406_2023_1553_MOESM1_ESM.docx]

**Supplementary Table 1:** Calculation of Pearson correlations to assess if relative (rel) volumes of anterior insula were associated with volumetric Ncl. accumbens, hypothalamus, pallidum externum / internum and lateral / medial habenula data *within the same heroin-addicted subjects*. *Annotation:* FDR, FDR-corrected p-value.

| **Brain region** | **Statistic** | **Anterior insula left (rel)** | **Anterior insula right (rel)** |
| --- | --- | --- | --- |
| Nucleus accumbens left (rel) | r | -0.032 | -0.137 |
|  | p | 0.913 (FDR:0.926) | 0.640 (FDR:0.882) |
|  | n | 14 | 14 |
| Nucleus accumbens right (rel) | r | -0.338 | -0.541 |
|  | p | 0.238 (FDR:0.713) | **0.046*** (FDR:0.549) |
|  | n | 14 | 14 |
| Hypothalamus left (rel) | r | 0.178 | -0.048 |
|  | p | 0.561 (FDR:0.748) | 0.876 (FDR:0.882) |
|  | n | 13 | 13 |
| Hypothalamus right (rel) | r | 0.230 | 0.083 |
|  | p | 0.429 (FDR:0.744) | 0.777 (FDR:0.882) |
|  | n | 14 | 14 |
| Pallidum externum left (rel) | r | 0.199 | 0.277 |
|  | p | 0.496 (FDR:0.744) | 0.338 (FDR:0.676) |
|  | n | 14 | 14 |
| Pallidum externum right (rel) | r | 0.398 | 0.368 |
|  | p | 0.158 (FDR:0.634) | 0.195 (FDR:0.585) |
|  | n | 14 | 14 |
| Pallidum internum left (rel) | r | 0.404 | 0.370 |
|  | p | 0.152 (FDR:0.634) | 0.192 (FDR:0.585) |
|  | n | 14 | 14 |
| Pallidum internum right (rel) | r | 0.516 | 0.429 |
|  | p | 0.059 (FDR:0.634) | 0.126 (FDR:0.585) |
|  | n | 14 | 14 |
| Lateral habenula left (rel) | r | 0.030 | -0.048 |
|  | p | 0.926 (FDR:0.926) | 0.882 (FDR:0.882) |
|  | n | 12 | 12 |
| Lateral habenula right (rel) | r | -0.329 | -0.321 |
|  | p | 0.297 (FDR:0.713) | 0.309 (FDR:0.676) |
|  | n | 12 | 12 |
| Medial habenula left (rel) | r | 0.234 | 0.066 |
|  | p | 0.463 (FDR:0.744) | 0.839 (FDR:0.882) |
|  | n | 12 | 12 |
| Medial habenula right (rel) | r | -0.091 | -0.184 |
|  | p | 0.779 (FDR:0.926) | 0.566 (FDR:0.882) |
|  | n | 12 | 12 |

**Supplementary Table 2:** Individual demographic, anterior and posterior insular volumes and neuronal cell count data of each subject.
